# Qualitative and Quantitative Analysis of Volatile Components of Zhengtian Pills Using Gas Chromatography Mass Spectrometry and Ultra-High Performance Liquid Chromatography

**DOI:** 10.1155/2016/1206391

**Published:** 2016-01-20

**Authors:** Cui-ting Liu, Min Zhang, Ping Yan, Hai-chan Liu, Xing-yun Liu, Ruo-ting Zhan

**Affiliations:** ^1^Research Center of Chinese Medicinal Resource Science and Engineering, Key Laboratory of Chinese Medicinal Resources from Lingnan of Ministry of Education, Joint Laboratory of National Engineering Research Center for the Pharmaceutics of Traditional Chinese Medicines, Guangzhou University of Traditional Chinese Medicine, Guangzhou, Guangdong 510006, China; ^2^School of Chinese Herbal Medicine, Guangzhou University of Chinese Medicine, Guangzhou, Guangdong 510006, China

## Abstract

Zhengtian pills (ZTPs) are traditional Chinese medicine (TCM) which have been commonly used to treat headaches. Volatile components of ZTPs extracted by ethyl acetate with an ultrasonic method were analyzed by gas chromatography mass spectrometry (GC-MS). Twenty-two components were identified, accounting for 78.884% of the total components of volatile oil. The three main volatile components including protocatechuic acid, ferulic acid, and ligustilide were simultaneously determined using ultra-high performance liquid chromatography coupled with diode array detection (UHPLC-DAD). Baseline separation was achieved on an XB-C18 column with linear gradient elution of methanol-0.2% acetic acid aqueous solution. The UHPLC-DAD method provided good linearity (*R* ^2^ ≥ 0.9992), precision (RSD < 3%), accuracy (100.68–102.69%), and robustness. The UHPLC-DAD/GC-MS method was successfully utilized to analyze volatile components, protocatechuic acid, ferulic acid, and ligustilide, in 13 batches of ZTPs, which is suitable for discrimination and quality assessment of ZTPs.

## 1. Introduction

In comparison with conventional fully porous particle columns, core-shell particle columns have higher column efficiency and sensitivity, achieve better separation, and remain constant over a wider linear range. In addition, the columns improve the speed of the mobile phase, reduce analysis time, and improve throughput. Moreover, the backpressure produced by the columns at optimum linear velocity is very low (<400 bar). So we take advantage of this new column to develop an efficient ultra-high performance liquid chromatography (UHPLC) method [1–4]. Gas chromatography mass spectrometry (GC-MS) has been employed in a broad range of analytical applications due to its high sensitivity and capacity to separate compounds effectively. GC-MS is commonly used to characterize and identify volatile organic compounds in complex mixtures [5–10]. GC-MS has become a popular and useful analytical tool in research on herbal medicines, especially in establishing chromatographic fingerprints for quality control of traditional Chinese medicines [11, 12], such as* Fructus Xanthii* [13].

Zhengtian pills (ZTPs) are Chinese patent medicine comprised of 15 medicinal herbs:* Caulis Spatholobi*,* Radix Angelicae Sinensis*,* Rhizoma Chuanxiong*,* Asari Radix et Rhizoma*,* Uncariae Ramulus Cum Uncis*,* Paeoniae Radix Alba*,* Radix Rehmanniae*,* Radix Angelicae Dahuricae*,* Radix Saposhnikoviae*,* Notopterygii Rhizoma et Radix*,* Persicae Semen*,* Carthami Flos*,* Radix Angelicae Pubescentis*,* Ephedrae Herba*, and* Aconiti Lateralis Radix Praeparata* [14]. ZTPs are used to treat tension headaches [15], headaches associated with spinal conditions, and premenstrual headaches. ZTPs are widely used in China to treat migraine headaches, a type of vascular headache [16, 17]. According to the Chinese Pharmacopoeia, paeoniflorin is the marker compound used for ZTP quality control [14].

In a previous study, HPLC was used to determine the major active components of ZTP, which included paeoniflorin, ferulic acid, prim-O-glucosylcimifugin, and 4′-O-beta-glucopyranosyl-5-O-methylvisamminol [18]; however, determination of these compounds is not sufficient for comprehensive quality control of ZTPs, which contain complex bioactive constituents, including volatile components of essential oils, alkaloids, flavonoids, and coumarin compounds. Several compounds from ZTPs ingredients, including protocatechuic acid from* Spatholobi Caulis*, and ferulic acid and ligustilide from* Rhizoma Chuanxiong* and* Radix Angelicae Sinensis*, have pharmacological activities, including antibacterial, anti-inflammatory, and analgesic effects, as well as protective effects on the cardiovascular system [19–21]. Therefore, an effective and reliable method capable of qualitative and quantitative analysis of the diverse bioactive constituents of ZTPs is required to ensure their safety and efficacy. While significant research has been conducted on the volatile components of* Rhizoma Chuanxiong* and* Radix Angelicae Sinensis*, such studies have mainly focused on their cardiovascular, cerebrovascular, neuroprotective, antinociceptive, and anti-inflammatory effects [22, 23], and the volatile components of the other ZTPs constituents have not been reported.

In this study, a combined UHPLC coupled with diode array detection (UHPLC-DAD)/GC-MS method was developed to simultaneously identify volatile organic compounds in ZTPs and qualitatively and quantitatively analyze 3 main bioactive constituents present in various ZTPs ingredients. The combined UHPLC-DAD/GC-MS method can be utilized to evaluate the quality of ZTPs.

## 2. Experimental

### 2.1. Reagents and Chemicals

HPLC grade anhydrous ether and methanol were purchased from Merck (Darmstadt, Germany). Deionized water for the samples and mobile phase were prepared using a Milli-Q50 SP Reagent Water System (Millipore, France). Reference standards for protocatechuic acid, ferulic acid, and ligustilide were obtained from the National Institute for Food and Drug Control (Beijing, China). The purity of all standards was at least 98%. Thirteen batches of ZTP were purchased from China Resources Sanjiu Medical & Pharmaceutical Co. Ltd. (Shenzhen, China). Methanol, acetic acid, petroleum ether, ethyl ether, and ethyl acetate were purchased from Baishi Chemical Industry Co. Ltd. (Tianjin, China) and of analytical grade. All solvents were filtered through 0.22 *μ*m membrane filters before analysis.

### 2.2. Sample Preparation

ZTPs were smashed into powder (40 mesh). For HPLC analysis, 3.0 g of pulverized ZTP was ultrasonically extracted at room temperature with 50 mL methanol for 0.5 h in a 250 mL triangular flask and dried. The ZTP residue was dissolved with 20 mL water and extracted 3 times with ethyl acetate (30, 30, and 20 mL). The ethyl acetate extracts were dried using an electrothermostatic water bath. Finally, the extract was reconstituted in 5 mL methanol. For GC-MS analysis, 3.0 g of pulverized ZTP was reflux-extracted twice for 1 h at 50°C with 20 mL ethyl acetate in a 250-mL triangular flask. The ethyl acetate extracts were dried and reconstituted in 5 mL ethyl acetate. All ZTP solutions were filtered through a 0.22 *μ*m nylon membrane filter.

### 2.3. GC-MS Analysis

GC-MS analysis was performed on an Agilent 7890/5975C-GC/MSD instrument (Agilent Technologies, USA) coupled with a HP-5MS fused silica capillary column (30 m × 0.25 mm × 0.25 *μ*m; Agilent Technologies, Santa Clara, CA, USA). The GC oven temperature was initially increased from 60°C to 120°C at a rate of 10°C/min, then elevated at a rate of 3°C/min up to 175°C, then increased at a rate of 5°C/min up to 205°C, then went up at a rate of 0.8°C/min up to 210°C, and then raised to 280°C at a rate of 5°C/min, held for 5 min, giving a total runtime of 55.583 min. 1 *μ*L volume of ethyl acetate extracts was injected into the GC. Helium carrier gas at a constant flow rate of 1.0 mL/min and a 30 : 1 split ratio were used simultaneously. Mass spectrometer was operated in full scan with an electron energy of 70 eV; interface temperature: 280°C; MS source temperature: 230°C; MS quadrupole temperature: 150°C. The scan range was from* m/z* 30 to 550.

### 2.4. HPLC Analysis

HPLC analysis was performed on a Thermo Ultimate-3000 system (Thermo Scientific, Waltham, MA, USA) coupled with a DAD. A Phenomenex Kinetex XB-C18 column (100 mm × 4.6 mm; 2.6 *μ*m i.d.; 25°C column temperature) with a guard column (2.1 mm × 4.6 mm, 2.6 *μ*m i.d.) was used (Phenomenex, Torrance, CA, USA). The mobile phase was 0.2% (v/v) aqueous acetic acid solution (A) and methanol (B). The linear gradient was as follows: 0–7 min, 10% B; 7–15 min, 10–15% B; 15–35 min, 15–40% B; 35–65 min, 40–50% B. The flow rate was 1.5 mL/min and the injection volume was 2 *μ*L.

### 2.5. HPLC Method Validation

All components were quantified using chromatograms obtained at 260 nm. The quantification was validated in terms of linearity, limit of detection (LOD), limit of quantification (LOQ), accuracy, and precision.

The stock solution containing the 3 markers was prepared and diluted to appropriate concentration ranges for the establishment of calibration curves. The calibration graphs were plotted after linear regression of the peak areas versus the corresponding concentrations. Good linear behavior was observed, with correlation coefficients (*r*) between 0.9992 and 0.9994. LOD and LOQ were determined at signal-to-noise (*S*/*N*) ratios of approximately 3 and 10, respectively. Recovery experiments were performed at medium levels. The concentrations of protocatechuic acid, ferulic acid, and ligustilide were 0.0204 mg/mL, 0.1027 mg/mL, and 0.22 mg/mL. This procedure was accordingly repeated for six replicates. The spiked samples were extracted, processed, and quantified in accordance with the methods described above. Recoveries varied from 100.18 to 103.39%, with RSDs from 0.50 to 2.36% (Table 1).

Precision was evaluated with the solution of sample 1209051H under the selected optimal conditions 6 times in 1 day to measure intraday variation. The RSDs of the precision results were in the range of 0.54–0.89%. Repeatability was confirmed with 6 different working solutions prepared from sample 1209051H. The RSDs of the repeatability results were in the range of 1.88–2.31%. The stability of the solutions was tested by injecting them into the apparatus at 0, 2, 4, 6, 8, 12, and 24 h. The RSDs of the stability results were in the range of 1.80–2.53%.

**Figure 1:**
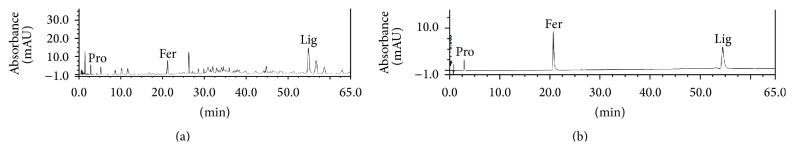
Representative HPLC chromatograms of mixed standards and the ZTP extract at 260 nm. (a) ZTP extract (sample 1209051H); (b) mixed standards of the 3 chemical constituents. Peaks: protocatechuic acid, ferulic acid, and ligustilide.

Moreover, specificity was investigated by comparing the chromatograms of mixed standards and the ZTP extract (Figure 1). According to the three-dimensional plot of the absorbance as a function of retention time and wavelength in the HPLC-DAD data for sample number 1209051H, no evidence of peak of impurities overlapping the markers was found.

This is the first simultaneous analysis of protocatechuic acid, ferulic acid, and ligustilide with acceptable linearity, precision, repeatability, and accuracy.

## 3. Results and Discussion

### 3.1. Optimization of the GC Method and Extraction

**Figure 2:**
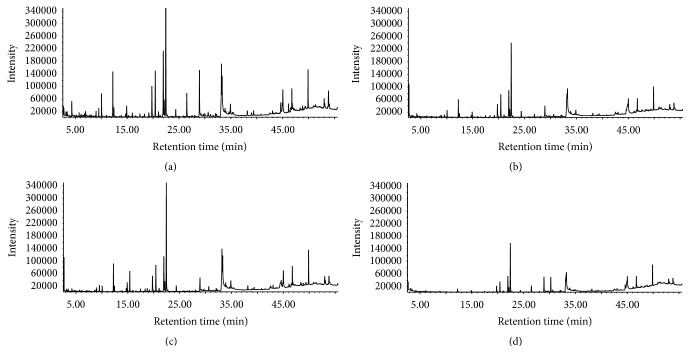
The total ion chromatogram (TIC) of ZTPs using different solvents and extraction methods: (a) ethyl acetate extraction by reflux method, (b) ethyl acetate extraction by ultrasonic wave, (c) ether extraction by ultrasonic wave, and (d) petroleum ether extraction by ultrasonic wave.

In this study, the extraction efficiencies of petroleum ether, ethyl ether, and ethyl acetate were compared. Total ion current (TIC) was greatest when ethyl acetate was used. Investigation of the extraction efficiencies of the reflux and ultrasonic methods indicated that total TIC was higher when the reflux method was used. The GC results for the different solvent extracts and methods are described in Table 2 and Figure 2. In the ethyl acetate solvent extracts, 22 components were identified by artificial analysis and computer retrieval, while relative content was determined by the area normalization method. The main components of the ethyl acetate solvent extracts were ligustilide (19.381%), oleic acid (10.012%), 9,12-octadecadienoic acid (9.346%), butylidene phthalide (5.055%), dibutyl phthalate (5.891%), xanthyletin (3.813%), methyl eugenol (3.833%), n-docosane (3.545%), asarinin (2.085%), and safrole (1.555%). In comparison to the ethyl acetate solvent extract samples, the ethyl ether extract samples contained a greater amount of 2,6-di-tert-butyl-p-cresol (2.667%), while the petroleum ether extract samples contained a greater amount of sulfur (3.993%).

### 3.2. Optimization of the HPLC Method and Extraction

Due to the existence of acidic components in the ZTP extraction, a small amount of acid was added to the mobile phase to ease ionization of these components, with the goal of improving peak shape and restraining peak tailing. Different concentrations of acetic acid, phosphoric acid, and formic acid were compared for this purpose. The results showed that all compounds could be baseline-separated when 0.2% aqueous acetic acid solution was added.

DAD detection was performed within a wavelength range of 190–400 nm. When the chromatograms and characteristic UV spectra of the 3 reference compounds were compared, it was found that the 3 active compounds had higher absorbance, better separation, and a steady baseline at 260 nm in comparison with the other tested wavelengths.

The extraction procedure was optimized prior to sample analysis. The samples (3.0 g each) were extracted with different volumes and percentages of methanol, as well as different volumes and percentages of ethanol, respectively. The optimum results were obtained using 50 mL methanol. Investigation of the dependence of the yield on the duration of the extraction (15, 30, and 60 min) showed that all of the investigated compounds were almost completely extracted when 30 min extraction was used.

### 3.3. Sample Analysis

The UHPLC-DAD/GC-MS method was applied to analyze 13 batches of ZTP (Tables 3 and 4). Protocatechuic acid content ranged from 6.07 to 17.30 *μ*g/g, ferulic acid content ranged from 79.59 to 115.90 *μ*g/g, and ligustilide content ranged from 79.59 to 388.69 *μ*g/g. The GC-MS results showed that the average relative contents of ligustilide, oleic acid, asarinin, safrole, 2-methoxy-4-vinylphenol, and 1,2-dimethoxy-4-(2-propenyl)benzene were 6.991%, 23.275%, 1.631%, 0.586%, 0.575%, and 0.574%, respectively. 3,5-Dimethoxytoluene was not detected. These results suggested that the contents of each component varied greatly among batches of ZTPs. The largest observed difference in content was nearly 5-fold, indicating large variations in the ZTP production process and/or incorrectly identified herbal sources.

The UHPLC-DAD/GC-MS method we established could detect as much volatile components as possible. Compared to previous study using a two-dimensional liquid chromatography coupled to mass spectrometry, there were more compounds identified by the GC-MS method, such as 1,2-dimethoxy-4-(2-propenyl)benzene, 2-methoxy-4-vinylphenol phenol, butylidene phthalide, oleic acid, and (Z,Z)-9,12-octadecadienoic acid ethyl ester, which were mainly from Rhizoma Chuanxiong [24]. It is very helpful for a comprehensive understanding of volatile components of ZTPs. At the same time, the established UHPLC-DAD method could determine protocatechuic acid, ferulic acid, and ligustilide simultaneously, which was very helpful and practicable for the quality control of ZTPs for companies. Since TCM is a complex system containing tens or even hundreds of different chemical constituents, the active compounds of most TCM still remain unknown. It is reported that molecular biochromatography was a novel strategy for the screening and analysis of biologically active compounds in TCM [25, 26]. Thus, in our further study, we will make an attempt to screen active compounds of ZTPs by using molecular biochromatography.

## 4. Conclusions

A UHPLC-DAD/GC-MS method was established for the comprehensive analysis of ZTPs that allowed separation of complex constituents in a short time. GC-MS provided accurate masses of protonated molecules, which were helpful for compound identification. The UHPLC-DAD/GC-MS method was successfully applied for simultaneous determination of 3 bioactive compounds in ZTP. The UHPLC-DAD/GC-MS method is readily available, rapid, and reliable. Therefore, the UHPLC-DAD/GC-MS method is suitable for routine analysis, original discrimination, and effective quality control of ZTPs. The amounts of protocatechuic acid, ferulic acid, and ligustilide in the ZTP batches varied considerably. Therefore, future studies should focus on evaluating the influence of bioactive constituent of ZTPs on their therapeutic effects using preclinical pharmacodynamic and clinical testing.

## Figures and Tables

**Table 1:** Regression equations, correlation coefficients, linearity ranges, LODs, LOQs, and recoveries of investigated compounds.

Analytes	Regression equation	*R* ^2^	Linearity range/*μ*g mL^−1^	LOD/*μ*g	LOQ/*μ*g	Recovery %	RSD %
Protocatechuic acid	*Y* = 38.403*X* + 0.0743	0.9994	4.1–28.6	0.0019	0.0037	100.68	0.35
Ferulic acid	*Y* = 13.889*X* + 0.3961	0.9992	80.2–561.4	0.025	0.074	100.98	0.56
Ligustilide	*Y* = 9.1687*X* + 1.1081	0.9994	110–770	0.044	0.084	102.69	0.28

**Table 2:** The relative content of components of ZTPs identified by GC-MS.

Peak number	Retention time (min)	Compound name	Molecular formula	*m*/*z*	Relative content (%)			
					Ethyl acetate refluxing	Ethyl acetate ultrasonic	Ether ultrasonic	Petroleum ether ultrasonic
1	2.808	3,5-Dimethoxytoluene	C_9_H_12_O_2_	152.19	0.637	1.718	1.372	1.703
2	4.312	Safrole	C_10_H_10_O_2_	162.19	1.555	—	—	—
3	10.059	2-Methoxy-4-vinylphenol phenol	C_9_H_10_O_2_	150.17	1.691	1.373	0.861	—
4	12.241	1,2-Dimethoxy-4-(2-propenyl)benzene	C_12_H_18_O_3_	210.27	3.833	2.839	3.238	0.768
5	12.442	3,4,5-Trimethoxytoluene	C_10_H_14_O_3_	182.22	0.755	0.571	0.689	—
6	14.901	Pentadecane	C_15_H_32_	212.41	1.319	1.269	—	0.795
7	15.384	2,6-Di-tert-butyl-4-methylphenol	C_15_H_24_O	220.35	—	—	2.667	—
8	20.422	Butylidene phthalide	C_12_H_12_O_2_	188.22	5.055	4.871	4.086	3.426
9	22.455	Ligustilide	C_12_H_14_O_2_	190.24	19.381	16.109	17.654	15.063
10	24.366	trans-Ligustilide	C_12_H_14_O_2_	190.24	1.007	1.566	1.06	0.79
11	26.502	1,2-Benzenedicarboxylic acid, bis(2-methylpropyl) ester	C_16_H_22_O_4_	278.34	2.724	0.719	—	1.881
12	28.946	Dibutyl phthalate	C_16_H_22_O_4_	278.34	5.891	3.172	5.891	5.193
13	30.251	Sulfur	S_8_	32.06	—	—	—	3.993
14	33.188	(Z,Z)-9,12-Octadecadienoic acid	C_18_H_32_O_2_	280.44	9.346	10.605	11.484	5.865
15	33.327	Oleic acid	C_18_H_34_O_2_	282.46	10.012	12.334	12.347	16.905
16	33.892	(Z,Z)-9,12-Octadecadienoic acid ethyl ester	C_20_H_36_O_2_	383.5	1.181	0.71	1.936	1.268
17	38.18	Tricosane	C_23_H_48_	324.63	0.706	0.678	0.789	0.888
18	44.619	(S)-8,8-Dimethyl-2-oxo-7,8-dihydro-2H,6H-pyrano(3,2-g)chromen-7-yl 3-methylbut-2-enoate	C_19_H_20_O_5_	328.36	1.464	1.209	2.318	1.962
19	45.03	8,8-Dimethyl-2H,8H-benzo[1,2-*b*:5,4-*b*′]dipyran-2-one	C_14_H_12_O_3_	228.24	3.813	4.629	4.166	7.711
20	46.766	Eicosane	C_20_H_42_	282.55	2.005	2.39	2.946	2.839
21	49.893	n-Docosane	C_22_H_46_	310.6	3.545	3.94	4.272	5.138
22	53.837	Asarinin	C_20_H_18_O_6_	354.35	2.085	1.9	1.671	2.01

**Table 3:** Relative constituent content determinations from 13 batches of ZTPs.

Sample number	3,5-Dimethoxytoluene (%)	Safrole (%)	2-Methoxy-4-vinylphenol phenol (%)	1,2-Dimethoxy-4-(2-propenyl)benzene (%)	Ligustilide (%)	Oleic acid (%)	Asarinin (%)
1209051H	—	0.744	0.321	0.267	4.97	26.166	0.758
1303002H	—	0.393	0.587	0.753	7.101	16.769	1.806
1212023H	—	0.321	0.393	0.438	5.747	32.844	1.084
1210019H	—	0.736	0.556	—	8.905	20.581	3.120
1212024H	—	0.556	0.420	0.309	5.903	34.815	2.163
1301023H	—	0.420	0.400	—	4.285	34.989	1.119
1302026H	—	0.400	0.753	—	7.558	18.568	1.334
1303036H	—	0.753	0.973	—	5.916	20.691	2.074
1304001H	—	0.973	0.803	—	10.845	19.593	1.560
1306019H	—	0.803	0.588	—	7.396	20.627	2.164
1306020H	—	0.588	0.458	—	5.725	18.016	1.344
1306027H	—	0.458	0.475	0.816	6.517	23.226	1.154
1309014H	—	0.475	0.744	0.858	10.012	15.691	1.523

**Table 4:** Determination of the contents of protocatechuic acid, ferulic acid, and ligustilide of 13 batches of ZTPs.

Sample number	Content (*μ*g/g)					
	Protocatechuic acid		Ferulic acid		Ligustilide	
	Mean	RSD (%)	Mean	RSD (%)	Mean	RSD (%)
1209051H	14.01	1.91	80.11	2.78	174.13	1.65
1303002H	14.43	1.26	79.59	1.42	79.59	2.52
1303002H	17.30	1.39	80.37	1.84	178.85	2.65
1210019H	8.36	0.82	78.06	2.02	363.42	0.39
1212024H	10.64	2.75	82.51	1.21	348.78	2.48
1301023H	12.21	1.25	86.42	0.88	252.37	2.57
1302026H	10.08	1.67	115.90	2.62	323.11	2.47
1303036H	6.07	2.51	84.98	3.00	388.69	1.05
1304001H	10.02	1.61	111.97	2.35	268.61	2.42
1306019H	9.62	1.61	86.68	1.32	303.62	0.81
1306020H	8.13	1.26	80.93	2.89	355.71	1.72
1306027H	9.71	0.37	93.14	0.49	260.75	0.52
1309014H	9.64	2.86	96.50	2.89	301.90	0.59
